# Supplementary material for: Outcomes of Patients with Lower Limb Loss after Using a Training Prosthesis: A Retrospective Case Series Study
Source: Healthcare (Basel). 2024 Feb 29;12(5):567. doi: 10.3390/healthcare12050567 (PMC10931137; doi:10.3390/healthcare12050567)
Supplement: Supplementary file 1 [file healthcare-12-00567-s001.zip › healthcare-2821363-supplementary.pdf]

Table S1. Characteristics of the matched population (n=90)

	Rehabilitation with TP (n=45)	Rehabilitation without TP (n=45)	*p*-Value[a]
**Sex** *(men)*	32 (71.1)	32 (71.1)	1
**Age** *(years)*	64 (55-70)	62 (55.5-70.5)	0.67
**BMI** *(kg/m²)*	23.4 (20.6-29.7)	26.5 (20.9-31.4)	0.22
**Vascular etiology** *(yes)*	2 (4.44)	5 (11.1)	0.43[b]
**Site of amputation**			0.23
Unilateral above-knee amputation *(yes)*	24 (53.3)	16 (35.5)	
Unilateral below-knee amputation *(yes)*	20 (44.4)	27 (60.0)	
Bilateral *(yes)*	1 (2.22)	2 (4.44)	
**Time between amputation and entrance into the rehabilitation center** *(days)*	17 (11-27)	20 (14-28)	0.20

TP: Training Prothesis; BMI: Body Mass Index

Qualitative variables are expressed in absolute (n) and relative (%) frequencies; quantitative variables are expressed in median and interquartile range (P25-P75)

[a]p-values obtained from the Mann-Whitney U test for continuous variables and the X^2 test for categorial variables

[b] p-value obtained from the exact Fisher test

Table S2. Outcomes associated with rehabilitation with or without TP in matched population (n=90)

	n	Rehabilitation with TP	Rehabilitation without TP	*p*-Value[a]
**Orientation at discharge**	90			0.33
Back to home		38 (84.4)	41 (91.1)	
To nursing facilities		7 (15.5)	4 (8.89)	
**Length of stay in rehabilitation center (days)**	88	70 (54-92)	99 (80-154)	<0.001
**Number of sockets required**	86			0.049
1 socket				

2 sockets		42 (97.7)	37 (86.1)	
**Interventions for size adaptation of the**		1 (2.32)	6 (13.9)	
**first socket of EP**	86	1 (0-2)	0 (0-1)	0.38
**Functional group**	71			0.78
Group 0		10 (24.4)	4 (13.3)	
Group 2		2 (4.88)	1 (3.33)	
Group 3		18 (43.9)	16 (53.3)	
Group 4		5 (12.2)	5 (16.7)	
Group 5		6 (14.6)	4 (13.3)	

TP: Training Prothesis; EP: Evaluation prothesis

Qualitative variables are expressed in absolute (n) and relative (%) frequencies; quantitative variables are expressed in median and interquartile range (P25-P75)

[a]p-values obtained from the Mann-Whitney U test for continuous variables and the X^2 test for categorial variables
